# Recent Advances in Prion Inactivation by Plasma Sterilizer

**DOI:** 10.3390/ijms231810241

**Published:** 2022-09-06

**Authors:** Akikazu Sakudo, Risa Yamashiro, Takashi Onodera

**Affiliations:** 1 School of Veterinary Medicine, Okayama University of Science, Imabari 794-8555, Ehime, Japan; 2 Laboratory of Biometabolic Chemistry, School of Health Sciences, University of the Ryukyus, Nishihara 903-0215, Okinawa, Japan; 3 Laboratory of Environmental Science for Sustainable Development, Department of Global Agricultural Science, Graduate School of Agricultural and Life Sciences, The University of Tokyo, Bunkyo-ku 113-8657, Tokyo, Japan; 4 Research Center for Food Safety, Graduate School of Agricultural and Life Sciences, The University of Tokyo, Bunkyo-ku 113-8657, Tokyo, Japan

**Keywords:** corona plasma, gas plasma, dielectric barrier discharge, hydrogen peroxide gas plasma, prion, radiofrequency

## Abstract

Prions, which cause transmissible spongiform encephalopathies (TSEs), are a notorious group of infectious agents with possibly the highest resistance to complete inactivation. Although various gas plasma instruments have been developed, studies on prion inactivation using gas plasma instruments are limited. Among them, the hydrogen peroxide gas plasma instrument, STERRAD^®^ (Advanced Sterilization Products; ASP, Johnson & Johnson, Irvine, CA, USA), is recommended for prion inactivation of heat-sensitive medical devices. However, STERRAD^®^ is not a plasma sterilizer but a hydrogen peroxide gas sterilizer. In STERRAD^®^, plasma generated by radio frequency (RF) discharge removes excess hydrogen peroxide gas and does not contribute to sterilization. This is also supported by evidence that the instrument was not affected by the presence or absence of RF gas plasma. However, recent studies have shown that other gas plasma instruments derived from air, nitrogen, oxygen, Ar, and a mixture of gases using corona, dielectric barrier, microwave, and pulse discharges can inactivate scrapie prions. As inactivation studies on prions other than scrapie are limited, further accumulation of evidence on the effectiveness of gas plasma using human-derived prion samples is warranted for practical purposes.

## 1. Prions and Hierarchy of Resistance

**Figure 1:**
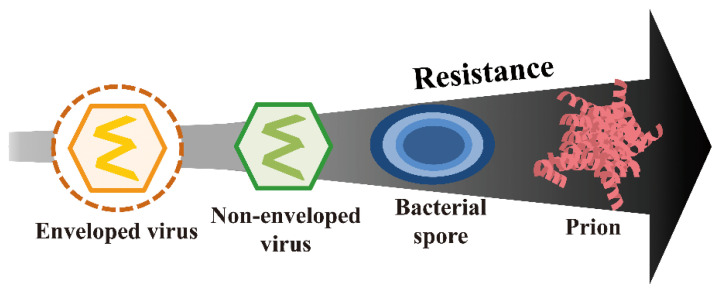
Prions exhibit the highest level of resistance to disinfection/sterilization. Bacterial spores, protozoal oocysts, and helminth eggs are highly resistant but display lower resistance than prions. Mycobacteria, small non-enveloped viruses, and fungal spores are moderately resistant. Vegetative bacteria, protozoa, fungi, algae, and large non-enveloped viruses are less resistant. Enveloped viruses are more susceptible. Illustrated based on the information sourced from the literature [1].

Microorganisms show varying resistance to disinfection/sterilization [1]. Figure 1 shows the resistance strengths of the various microorganisms and prions. Although prions are not strictly considered to be living organisms, they have been included in the figure for the comparison of their resistance with that of microorganisms. Prions represent the highest level of resistance, while enveloped viruses are susceptible to various disinfection and sterilization treatments. Sterility is achieved when the number of bacterial spores reaches a sterility assurance level (SAL) of 10^−6^ or less. Therefore, since prions are more resistant than bacterial spores, they require special treatment and may remain infectious even after sterilization.

Prion diseases, such as Creutzfeldt–Jakob disease (CJD) and bovine spongiform encephalopathy (BSE), are caused by prions [2]. Prion diseases can be classified according to their etiology as inherited, acquired, or sporadic [3]. Inherited prion diseases are caused by mutations in the prion protein (PrP) gene and include familial CJD (fCJD), Gerstmann–Sträussler–Scheinker syndrome (GSS), and fatal familial insomnia (FFI) [4]. Acquired prion diseases result from exposure to prion agents. For example, iatrogenic CJD (iCJD) has been confirmed following dural transplantation, pituitary growth hormone injection, and corneal transplantation [5,6,7], while variant CJD (vCJD) is derived from BSE-contaminated bovine materials [8]. Sporadic CJD (sCJD) accounts for approximately 85% of all human prion diseases [2]. However, unlike with inherited and acquired prion diseases, the cause of sCJD remains unknown [9].

**Figure 2:**
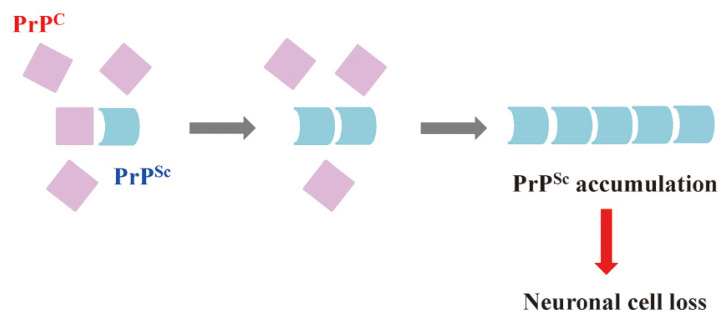
PrP^Sc^ accumulation is the cause of prion diseases. During prion infection, PrP^Sc^ is converted from PrP^C^. PrP^Sc^ accumulates in the brain, resulting in neuronal cell loss and, finally, death. PrP^Sc^: abnormal isoform of prion protein; PrP^C^: cellular isoform of prion protein.

Prions are mainly composed of abnormal prion proteins (PrP^Sc^) [2]. Neurons generally express a large amount of cellular prion protein (PrP^C^); however, in the diseased state, PrP^C^ can be converted to PrP^Sc^ by an unknown mechanism (Figure 2). The subsequent accumulation of PrP^Sc^ in cells leads to neuronal cell loss and death due to prion disease development [2].

## 2. Conventional Methods for Prion Inactivation

Common sterilization methods used for bacteria and viruses, such as high-pressure steam sterilization (121 °C, 20 min), ultraviolet irradiation, formalin fixation, and gamma irradiation, are insufficient to eliminate the infectivity of prions [10]. To control or minimize the risk of prion contamination, several methods, such as incineration, prolonged autoclaving (134 °C, 18 min), 1–2 N sodium hydroxide (NaOH) exposure for 1 h, and sodium hypochlorite (20,000 ppm available chlorine) treatment for 1 h, are available [10]. However, these are not practical for the treatment of reusable medical devices in clinical conditions because these procedures are time-consuming and may damage medical devices.

There are several widely adopted recommendations for prion inactivation. Reusable medical devices could become contaminated with prions and may transmit a prion disease upon contact with possible infectious tissues from previous patients (categorized into high, low, and no detectable infectivity) [11,12]. These devices should be subjected to treatment according to the presently available guidelines [13,14].

For example, the Japanese government requires consecutive serial measures for the effective inactivation of prions [15], referring to previous studies [16,17,18,19] and manuals from the French Government [20]. Guidelines recommend effective prion inactivation methods: (1) Wash thoroughly to remove attached prions on the surface of machines and instruments (washer-disinfectors; WDs); (2) use WDs using hot alkaline solution to denature the chemical composition of prions; (3) use WDs for heat-resistant and alkali-resistant instruments or machines, if these materials can be dipped into the WD solution; (4) a pre-vacuum autoclave is useful for heat-resistant instruments; and (5) hydrogen peroxide gas plasma can be used for heat-sensitive medical devices. However, it should be noted that inactivation programs must follow ISO 17,664 standards [21]. The guidelines [15] recommend the following procedures for prion inactivation of surgical devices used for high-risk surgical procedures: (1) Use WDs using alkaline solution + pre-vacuum autoclave at 134 °C, 8–10 min; (2) wash thoroughly using appropriate cleaners + pre-vacuum autoclave at 134 °C, 18 min; (3) wash using alkaline solution under appropriate concentration and temperature + hydrogen peroxide gas plasma sterilization (appropriate sterilization program, where prion inactivation should be confirmed using an equivalent model). However, these procedures include more than two sterilization steps and are thus time-consuming and sometimes impractical. Continuous inactivation is necessary to achieve more effective standards for prion inactivation.

Recently, plasma technology, which has attracted considerable attention due to its ability to inactivate pathogens, has also been studied as a novel method for prion inactivation [22,23]. In particular, the use of hydrogen peroxide gas plasma sterilization is recommended as a preventive measure against prion infection in heat-sensitive medical devices [15]. In contrast, the mechanism of prion sterilization using this method seems to be attributed to hydrogen peroxide gas but not to plasma because the contribution of plasma has not been observed [24,25,26]. Plasma instruments using gases other than hydrogen peroxide gas have also recently succeeded in prion inactivation [27,28,29,30]. Next, we introduce the fundamentals of plasma and recent studies on prion inactivation.

## 3. Fundamentals of Plasma

**Figure 3:**
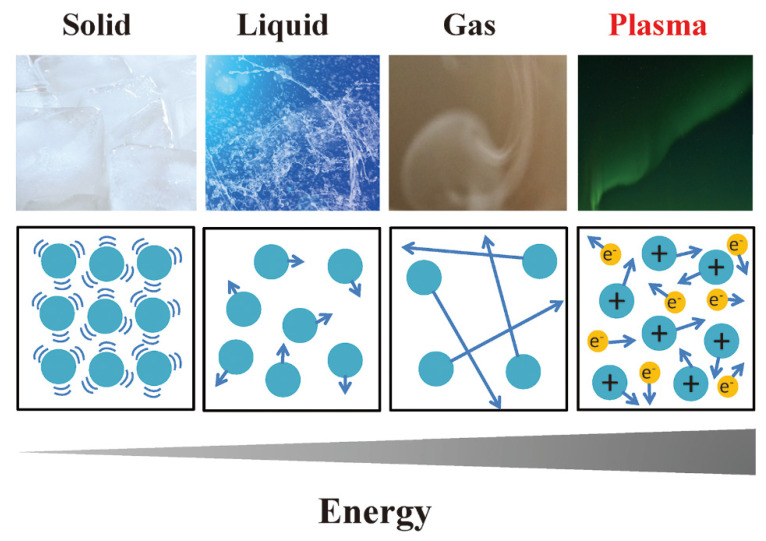
Plasma is the fourth state of matter. When energy is added to a solid, the state changes to liquid. Further energy addition transforms the liquid into a gas. When additional energy is applied to gaseous matter, the state transforms further into an ionized gas known as “plasma” or “gas plasma” (red).

Matter can exist in four states: solid, liquid, gas, and plasma. Although the most common state of matter in the universe is plasma, this was not discovered until 1927. Langmuir named this new state of matter “plasma” because he thought its properties were similar to those of blood plasma in that it can change its shape according to the container [31,32]. When energy is applied to matter, the movement of its atoms increases, resulting in a state change from solid to liquid and then from liquid to gas. When more energy is applied, the gas becomes ionized and electrons are liberated from the atoms to generate ionized gas, which is defined as plasma or gas plasma [33,34]. Plasma is the fourth state of matter after solids, liquids, and gases (Figure 3). In plasma, the number of positively charged particles is almost equivalent to that of negatively charged particles, indicating that plasma is electrically neutral at macroscopic scales. In nature, examples of plasma include the sun, aurorae in the upper atmosphere of the Earth, and lightning [35,36]. Plasma is also used in our daily lives, such as in fluorescent lights and neon signs [37,38].

To date, plasma technology has been utilized mainly in the field of engineering (plasma etching and nuclear fusion), which utilizes high-energy plasmas [39,40]. However, in recent years, considerable resources have been directed toward analyzing the potential biological applications of plasma, especially in innovative medical treatments [41,42,43]. Currently, plasma sterilization is becoming increasingly commercially available and constitutes the main purpose of plasma use in the medical field [22].

## 4. Sterilization/Disinfection/Inactivation by Plasma

Sterilization of pathogens attached to medical devices is important to prevent secondary infections. Currently, sterilization or disinfection is performed by autoclaving or exposure to gamma rays, ultraviolet light, or ethylene oxide gas [10]. As each of these procedures has its own advantages and disadvantages, it is necessary to select a suitable sterilization and disinfection method for specific circumstances [22]. Plasma technology is attracting attention as a novel sterilization/disinfection method because it facilitates low-temperature high-throughput treatment that is potentially environmentally friendly. Plasma demonstrates efficacy against various pathogens, including bacterial spores and prions, which are highly resistant to standard sterilization techniques [44]. Since plasma elicits an inactivation effect that is restricted to the exposed area, it is possible to inactivate the surface of the exposed object without affecting its interior. Therefore, plasma technology is suitable for surface disinfection and sterilization. Plasma is composed of reactive chemical species, including electrons, ions, neutral molecules, atoms, and charged species [33,45]. Furthermore, radiation in the UV/visible/near-infrared region can be emitted during plasma generation. Currently, sterilization and disinfection by plasma are thought to result from the combined effect of reactive chemical species present in plasma [22]. It is speculated that reactive chemical species in the plasma react with microorganisms and cause oxidation of biomolecules, such as proteins, nucleic acids, and lipids [22,46,47,48,49,50,51,52,53]. It has also been proposed that inactivation mechanisms depend on the conditions used for plasma generation, for example, type of feed or inlet gas, voltage setting, and discharge type. Indeed, spore death patterns have been shown to differ depending on the feed/inlet gas used for plasma generation [22,54,55]. 

## 5. Prion Inactivation Using Hydrogen Peroxide Gas Plasma

Most studies on prion inactivation by gas plasma use STERRAD^®^ (Advanced Sterilization Products; ASP, Johnson & Johnson, Irvine, CA, USA), which is a hydrogen peroxide gas plasma sterilizer that is widely used to sterilize heat-sensitive medical instruments [56,57]. STERRAD^®^ generates gas plasma by radio frequency (RF) discharge, which is generated by an RF power supply at 13.56 MHz (Table 1). The RF discharge generates a large volume of highly dense plasma with minimal heating under low atmospheric pressure. Therefore, RF discharge is often chosen as a sterilizer to disinfect fragile materials that are less likely to be damaged by treatment. Furthermore, the type of plasma generated can be selected based on the object being irradiated. However, in the case of STERRAD^®^, RF plasma is only used for the removal of hydrogen peroxide gas and not for sterilization [24,25,26]. The presence or absence of RF gas plasma did not affect the sterilization efficacy of the instrument [24]. Therefore, in terms of microcidal mechanisms, STERRAD^®^ may be categorized as a hydrogen peroxide gas sterilizer but not a hydrogen peroxide gas plasma sterilizer.

In addition to hydrogen peroxide gas, peracetic acid [58], ozone (O_3_), and chlorine dioxide (ClO_2_) [59] have been used for plasma sterilization. The effectiveness of these systems as sterilizers has been proven. However, these instruments have limitations in terms of toxic residues and material compatibility. Therefore, the US Food and Drug Administration (FDA) does not recommend the use of these plasma-based systems for clinical purposes due to toxicity and safety issues [60,61].

**Table 1:** The representative results of prion inactivation by hydrogen peroxide gas plasma.

Prions	Instruments	Plasma Types (Total Process Time for the Treatment)	Sample Types	Main Results of Inactivation	Reference
Scrapie (263 K)	Hydrogen peroxide gas plasma (STERRAD^®^)	RF plasma (for degradation of residual hydrogen peroxide)	Prion-contaminated steel wire	KOH-based detergent—70 °C for 10 min + STERRAD^®^ 100S GMP (59% hydrogen peroxide gas, 50 °C, 16 min, two cycles). Survival rates of animals were 100% (hamster), while those of untreated controls was 0% (incubation time: >397 days vs. control 81 days)	[62]
Scrapie (263 K)	Hydrogen peroxide gas plasma (STERRAD^®^)	RF plasma (for degradation of residual hydrogen peroxide)	Prion-contaminated steel wire	STERRAD^®^ NX (90% hydrogen peroxide gas, 53 °C, 7 min). More than 5 log reduction of prion infectivity. Survival rates of animals were 100% (incubation time: 528 days vs. control 85 days)	[63]
Scrapie (263 K)	Hydrogen peroxide gas plasma (STERRAD^®^)	RF plasma (for degradation of residual hydrogen peroxide)	Prion-contaminated steel wire	STERRAD^®^ NX (90% hydrogen peroxide gas, 53 °C, 7 min). More than 5 log reduction of prion infectivity. Survival rates of animals were 100% (incubation time: 570 days vs. control 78 days)	[64]
Scrapie (Chandler)	Hydrogen peroxide gas plasma (RENO-S130)	DBD plasma (for sterilization) and corona plasma (for degradation of residual hydrogen peroxide)	Prion-contaminated cover glass	RENO-S130 (50% hydrogen peroxide, below 60 °C; non-lumen mode: 28 min; Eco mode: 45 min). Survival rates of animals were 83% (both non-lumen and Eco mode) after injection with plasma-treated prions. Survival of untreated controls was 0%	[65]

RF: radio frequency; DBD: dielectric barrier discharge.

Next, we performed prion inactivation studies using STERRAD^®^. In an earlier study, the effectiveness of STERRAD^®^ NX against 263K scrapie prion was confirmed by Yan’s group [63]. This group also reported that STERRAD^®^ 100S GMP (59% hydrogen peroxide gas, 50 °C, 6 min) did not achieve sufficient prion inactivation, but their two-cycle treatment using STERRAD^®^ 100S GMP combined with KOH treatment at 70 °C for 10 min was effective [62].

Rogez-Kreuz et al. reported that a stainless steel wire contaminated with 263K scrapie prion could be efficiently inactivated using STERRAD^®^ NX or STERRAD^®^ 100S [64]. The results demonstrated that no mice (0/8) developed prion disease after the implantation of a prion-contaminated steel wire following STERRAD^®^ NX treatment (90% hydrogen peroxide gas, 53 °C, 7 min), suggesting that the infectious titer was reduced by at least 5 to 6 logs. On the contrary, STERRAD^®^ 100S (59% hydrogen peroxide gas, 50 °C, 20 min) showed almost no inactivating activity against the scrapie prions.

The differences in effectiveness between STERRAD^®^ NX and STERRAD^®^ 100S may be due to variations in the concentration of hydrogen peroxide gas used. This indicates that specific treatment conditions, such as gas concentration, incubation temperature, and incubation time, are important for prion inactivation. Therefore, if there is no previous information regarding prion inactivation using the same sterilization conditions (hydrogen peroxide concentration, incubation temperature, and incubation time) in a peroxide gas plasma sterilizer, experiments should be performed using each instrument of the same model to confirm prion inactivation activity. 

**Figure 4:**
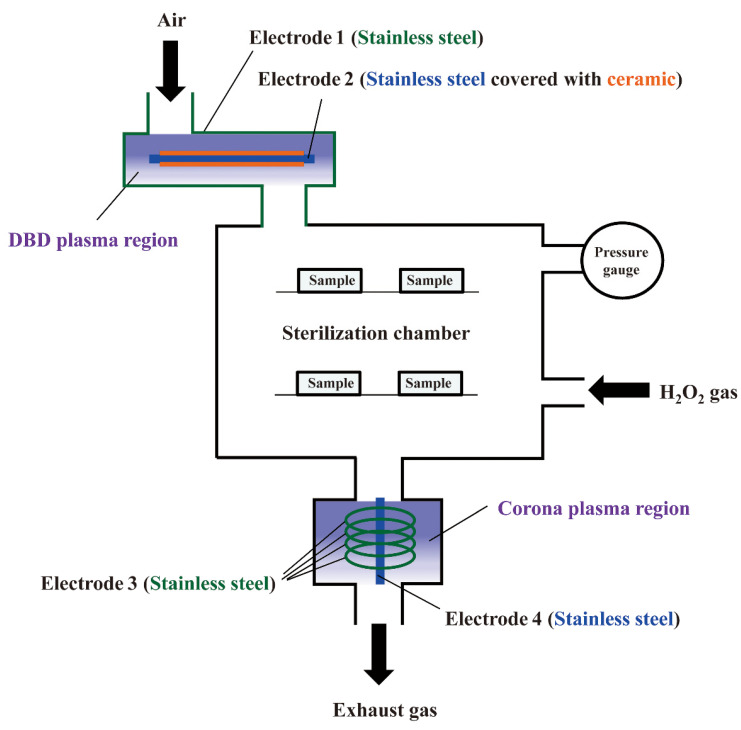
Schematic presentation of RENO-S130, a hydrogen peroxide gas plasma instrument used for prion inactivation experiments. Treatment modes include non-lumen mode (28 min) or Eco mode (45 min). In both treatment modes, hydrogen peroxide gas derived from 50% hydrogen peroxide was used. Air was injected at a flow rate of 5–10 L/min, as shown. There are two plasma regions, including the dielectric barrier discharge (DBD) plasma region and the corona plasma region. Electrode 1 (stainless steel chamber wall) and electrode 2 (stainless steel covered with ceramic) are located in the DBD plasma region. The DBD plasma region is located prior to the sterilization chamber box. In the corona plasma region, residual hydrogen peroxides are degraded and removed. H_2_O_2_: hydrogen peroxide. Reproduced from [65], published under an open access Creative Commons CC BY 4.0 license.

As mentioned above, STERRAD^®^ generates plasma using RF discharge. Recently, the authors examined other hydrogen peroxide gas plasma instruments to evaluate prion inactivation [65]. RENO-S130 (Renosem Co., Ltd., Bucheon-si, Korea) utilizes hydrogen peroxide gas plasma similar to STERRAD^®^ but does not use RF plasma [66]. Alternatively, RENO-S130 uses two types of plasma generation methods: corona discharge and dielectric barrier discharge (DBD) for gas plasma generation (Figure 4). 

To generate corona discharge, highly curved small-diameter stainless steel wires were used in the RENO-S130. A high voltage was applied using an alternating power supply (peak-to-peak (V_pp_) voltage of 34 kV; frequency of 15 kHz) to produce a strong electric field that was concentrated at the highly curved electrode surface, creating an uneven electric field surrounding the electrode. The concentrated electric field induces ionization of neutral gas in the immediate vicinity of the curved electrode surface. Finally, the local dielectric breakdown generates a corona discharge plasma.

The DBD can be generated by applying alternating high-voltage pulses using an alternating power supply (V_pp_ of 34 kV; frequency of 15 kHz). One electrode (electrode 2) was covered by a solid, ceramic, insulating material, while the other electrode (electrode 1) was uncovered. The ceramic insulator covering the electrodes suppressed the arc discharge. Thus, the temperature of the ions and neutral particles did not increase due to the short time required for DBD plasma generation. 

The authors succeeded in prion inactivation using hydrogen peroxide gas plasma and RENO-S130 against scrapie prions [65]. RENO-S130 is composed of a DBD plasma region for sterilization and a corona plasma region for the removal of residual hydrogen peroxide. There are two types of process modes: the non-lumen mode (28 min process time) and the Eco mode (45 min process time). Samples obtained from Chandler scrapie prion-infected mouse brain homogenates on glass surfaces were subjected to treatment. Thereafter, PrPres (proteinase K-resistant PrP), an index of PrP^Sc^, as well as total PrP, including both PrP^Sc^ and PrP^C^, in RENO-S130 treated and untreated samples were examined by western blot analysis using an anti-PrP antibody. The results showed that the levels of PrPres and total PrP were degraded by RENO-S130 treatment under both non-lumen and Eco mode conditions.

Furthermore, a mouse bioassay showed that treatment of prions with RENO-S130 (both non-lumen and Eco modes) increased the survival rates of mice (Table 2) and prolonged mouse survival time (Table 3). Significant differences in survival curves analyzed using log-rank tests were observed between the RENO-S130 (non-lumen or Eco mode)-treated and untreated groups in the mouse bioassay [65]. Results of protein misfolding cyclic amplification (PMCA) also supported the evidence of prion inactivation using the non-lumen or Eco mode RENO-S130 treatment.

The sterilization activity of RENO-S130 appears to be related to the co-generation of chemicals other than hydrogen peroxide gas. Ozone generated by DBD plasma enters the sterilization chamber region, and 10–50 ppm of ozone gas was detected using an ozone analyzer UV-100 (Eco Sensors Division of KWJ Engineering Inc., Santa Fe, NM, USA) in the RENO-S130 sterilization chamber box during operation [65]. Ozone synergistically enhances the prion inactivation efficiency of hydrogen peroxide gas [67]. Therefore, enhanced prion inactivation may be attributed to the ozone generated by DBD plasma. 

Together, both STERRAD^®^ and other hydrogen peroxide gas plasma sterilizers inactivate prions and may be used for the sterilization of prion-contaminated heat-sensitive medical devices since the process temperature remains below 60 °C. In STERRAD^®^, hydrogen peroxide gas is the main effector as a sterilizer, while gas plasma itself is the main contributor to the degradation of residual hydrogen peroxide but not to sterilization. In contrast, RENO-S130 generates ozone via DBD plasma, which contributes to sterilization, whereas corona plasma degrades residual hydrogen peroxide. Thus, enhanced inactivation efficiency is anticipated due to the concomitant generation of ozone. 

## 6. Inactivation of Prions Using Plasma Derived from Air, Nitrogen, Oxygen, Ar, and Their Mixtures

Although prion inactivation studies using gas plasma in addition to STERRAD^®^ are limited, some studies have used gas plasma instruments derived from air, nitrogen, oxygen, Ar, and their mixtures [22,27,28,29,30]. The electrical discharge methods of plasma generation used for prion inactivation include corona, RF, microwave, DBD, and pulse discharges (Table 4).

Jalák et al. [28] reported that prions (RML5, Rocky Mountain Laboratory scrapie strain 5) could be inactivated by open air-derived corona plasma. To measure prion infectivity after the plasma treatment, they used a cell infectivity assay involving cell blotting using CAD5 cells, which are derived from Cath.a-differentiated (CAD) cells and are susceptible to RML5. In the assay, the staining intensity, indicating cellular infection by prions, appeared to decrease after plasma exposure, suggesting that the infectivity of prions was reduced by plasma treatment.

In addition, Baxter et al. published a report on the treatment of a 263 K scrapie prion using RF plasma generated from an Ar/O_2_ gas mixture [29]. In their experiments, stainless steel spheres were contaminated with scrapie prions and then treated with RF plasma for 1 h. A bioassay was conducted based on intraperitoneal inoculation of recovered prions into hamsters. The results demonstrated that hamsters injected with untreated spheres showed clinical symptoms at 92 days, while hamsters injected with plasma-treated spheres survived for more than 466 days. These results suggest that Ar/O_2_ RF plasma reduces 263K prion infectivity levels below the limit of detectability.

In addition, the BIODECON group reported the treatment of prion-contaminated steel wires or silk sutures using low-pressure microwave-derived inductively coupled plasma (ICP) of Ar and Ar mixed with various other gases [30]. In this microwave discharge by electromagnetic radiation with a frequency of 13.56 MHz, plasma can be generated using ICP antennas with shielding. A large-diameter plasma discharge was generated under a low gas pressure (0.1 to 20 Pa). Steel wires were used to model a heat-resistant surface, whereas silk sutures were used to represent a heat-sensitive surface. Both mouse and hamster bioassays showed that Ar plasma efficiently inactivated prions compared with mixed Ar/N_2_ plasma [30]. However, the authors discussed that Ar plasma showed significant surface heating, which may contribute to prion inactivation. Maximum inactivation by the plasma instrument was achieved using Ar/O_2_. 

**Figure 5:**
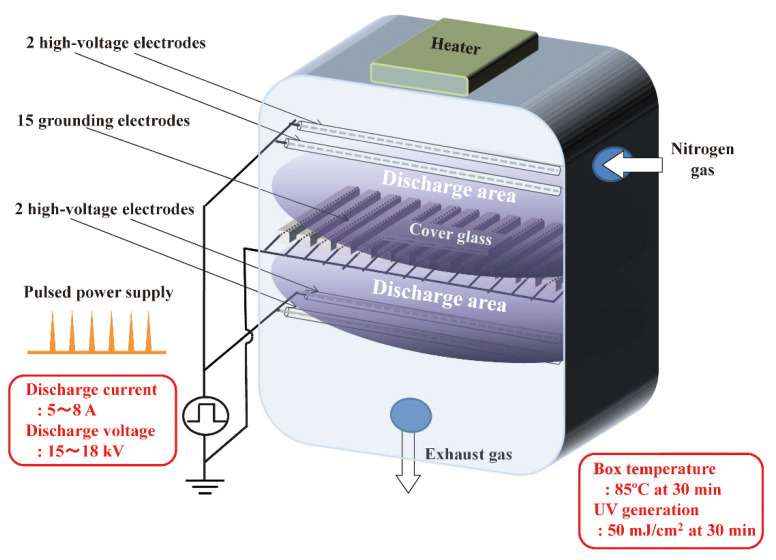
Schematic presentation of bipolar and low-pressure plasma–triple-effect sterilization (BLP-TES) instrument. BLP-TES is a nitrogen gas plasma instrument used for prion inactivation experiments. This instrument has 15 grounding electrodes located centrally between the two upper and two lower high-voltage electrodes (four high-voltage electrodes in total). Chamber box pressure is controlled by a pressure gauge and maintained at 0.5 atmospheres during the electrical discharge at 1.5 kilo pulse per second (kpps) and the discharge current of 5–8 A as well as the discharge voltage of 15–18 kV. The temperature of the chamber box was 85 °C at 30 min, while the UV intensity was 50 mJ/cm^2^ [49]. Reproduced from [27], published under an open access Creative Commons CC BY 4.0 license.

In the early 2010s, nitrogen gas plasma was successfully generated by pulse discharge plasma using a short-pulsed and highly repetitive high-voltage pulse power source with a bipolar and low-pressure plasma–triple-effect sterilization (BLP-TES) device (NGK Insulators, Ltd., Nagoya, Japan) [68]. Pulse discharge was generated by applying a high-voltage pulse for a short time using an induction energy storage (IES) circuit with a static induction (SI) thyristor to nitrogen (Figure 5). In pulse discharge, the heavy ions are not accelerated, unlike the electrons, enabling a stable discharge to occur relatively easily, even under nearly atmospheric pressure (approximately 0.5 atm). Since a short duration voltage is applied, the transition into an arc discharge can be prevented in this device.

Interestingly, BLP-TES has shown virucidal and bactericidal activity [47,48,49,50,51,52]. BLP-TES was found to be superior to STERRAD^®^ with respect to its endotoxin inactivation efficiency [48,54,69]. The susceptibility of amino acids to nitrogen plasma differs the heating [68]. The authors speculated that nitrogen gas plasma also changes the amino acids of proteins. The susceptibility of amino acids to nitrogen plasma-induced alterations varies [68]. The authors treated bovine serum albumin (BSA) with nitrogen gas plasma and did not detect fragmented BSA after gas plasma exposure. BLP-TES treatment of BSA resulted in increased α-helices and reduced β-sheets in the protein [68]. This suggests that the alterations induced by this treatment are due to modifications rather than degradation. 

Based on this information, we considered the potential use of BLP-TES for prion inactivation [27]. Aliquots of mouse brain homogenates infected with Chandler scrapie prions were spotted onto cover glasses and subjected to BLP-TES treatment. Untreated control prion samples were prepared in the same way but without plasma treatment. PMCA showed that BLP-TES treatment at 1.5 kpps for 15 or 30 min reduced the in vitro propagation levels of PrPres. Furthermore, mice injected with prions treated with plasma for 30 min showed longer survival than mice injected with untreated control prions (Table 5). These results indicate that BLP-TES treatment decreased prion infectivity. Taken together, these results suggest that BLP-TES treatment can inactivate Chandler scrapie prions by reducing their propagation activity and infectivity.

Together, these studies have shown that prions can be inactivated by exposure to plasma generated by various discharge methods and gas sources. 

## 7. Conclusions

Several scientists have confirmed the usefulness of hydrogen peroxide gas plasma and other gas plasmas derived from feed/inlet gases, such as air, nitrogen, oxygen, Ar, and their mixtures for prion inactivation [22,55]. The authors also used nitrogen gas plasma sterilizer BLP-TES and hydrogen peroxide gas plasma sterilizer RENO-S130 against scrapie prions and confirmed their effectiveness as sterilizers for prion inactivation [27,65].

Prions are known to bind strongly on steel surfaces [70,71]. Therefore, experiments have been performed using prion-contaminated steel wires to represent certain surgical instruments, as well as other surface materials, such as glass, as surrogates for some medical devices. However, apart from these materials, there are various surface materials used in medical devices, which may influence the inactivation effect. Generally, pretreatment washing of medical devices using enzymes or alkaline detergents is introduced before the sterilization process and will achieve a 4–6 log reduction in microorganisms and proteins [72]. Hence, pretreatment and exposure to gas plasma instruments during the sterilization process may enhance the total reduction in prion infectivity. Further studies on the effect of surface materials in combination with washing are required for each gas plasma sterilizer for practical use as a prion decontamination tool for medical devices.

To date, only scrapie prions or mouse-adapted prions have been used for prion inactivation studies, whereas there are a variety of prions in humans and animals [2]. It should be emphasized that the resistance of prions to inactivation differs among species or sources. For example, human CJD prions can be 100,000 times more difficult to inactivate than scrapie prion Sc237 during acidic sodium dodecyl sulfate (SDS) treatment [73]. Similarly, the BSE prion is reportedly 1000 times more resistant than the mouse-passaged BSE strain [74]. Therefore, we should note that extrapolation from rodent-passaged prion strains to human and bovine prion strains is unreliable. Even if human-derived prion strains are used for experiments, the data are insufficient because a variety of prion diseases produce various types of prions. Resistance may vary depending on the precise nature of the prion (i.e., vCJD, sCJD, GSS, or FFI). Even in CJD, type 1 and type 2 PrP^Sc^ are biochemically characterized [75]. sCJD can develop using six genotype/PrP^Sc^ combinations (MM1, MM2, MV1, MV2, VV1, and VV2) and result in five major strains of sCJD: MM1/MV1, MV2/VV2, MM2 cortical (MM2c), MM2 thalamic (MM2t), and VV1 [76,77,78,79]. These variations probably influence their individual resistance. Therefore, further studies on the effectiveness of prion inactivation using hydrogen peroxide gas plasma, as well as other gas plasmas, against all types of prions are necessary. In particular, experiments using clinically derived prion strains are warranted before the practical use of sterilizers for prion inactivation.

Plasma inactivating mechanisms depend on the type of feed/inlet gases used to generate the plasma. In the case of nitrogen gas plasma, three major mechanisms, including the action of reactive chemical species, ultraviolet (UV) irradiation exposure, and the effect of electric fields, are thought to be involved [54]. In most cases, the reactive chemical species produced from oxygen and nitrogen are the principal inactivating factors [45,54]. Etching also contributes, particularly in oxygen plasmas [80]. Thus, in the case of plasma derived from oxygen, air, and oxygen mixtures, the etching effect would contribute. Shrinking was observed in oxygen plasma-treated bacterial spores but not in nitrogen gas plasma-treated spores [81,82]. Therefore, as the inactivating mechanisms depend on the gas types and target microorganisms, each mechanism should be clarified for each combination of gas plasma instruments and target microorganisms. Clarification of the inactivating mechanisms would also contribute to enhancing the inactivation efficiency.

PrP^C^ is composed of unglycosylated, mono-glycosylated, and di-glycosylated forms [83], with the di-glycosylated form being the dominant form [83]. In addition, glycosylation can modulate the conversion of PrP^C^ into PrP^Sc^ in vitro [84]. Interestingly, a study using a thioreductant, dithiothreitol (DTT), indicated that the increased rate of disulfide bridges in PrP^C^ favors the formation of the unglycosylated form of PrP^C^ [85]. These results imply that redox state changes may affect PrP^C^ glycosylation or their processing, resulting in changes in the PrP^C^→PrP^Sc^ conversion efficiency. As reactive chemical species, which are the main inactivating factors produced during the generation of gas plasma, affect the redox state, gas plasma treatment may influence the glycosylation ratio of PrP^C^, resulting in the change of the PrP^C^→PrP^Sc^ conversion efficiency. However, it remains unclear whether PrP^C^ glycosylation status and PrP^Sc^ conversion efficiency are related in vivo. Thus, further studies on the relationship are required to elucidate the conversion mechanisms and explore the potential adverse effects of gas plasma on PrP^C^.

In recent years, the evidence that protein misfolding disorders, such as Alzheimer’s disease (AD), Parkinson’s disease (PD), amyotrophic lateral sclerosis (ALS), metabolic diseases, and cancer, share certain mechanisms of molecular pathogenesis, has been considered [86]. Self-propagating aggregation and cell-to-cell spreading, both in vitro and in vivo, have been observed in these protein misfolding disorders [87,88]. However, as human epidemic infectious diseases cause neither AD nor PD, nor any other protein misfolding disorder, they are distinct from prion diseases [89]. Thus, it is controversial as to whether these aggregates should be termed “prions,” or as other terms, such as “prionoids” or “prion-like aggregates” [89,90]. It should be noted that the effect of gas plasmas may vary among “prions”, “prionoids”, or “prion-like aggregates” derived from different species and neurodegenerative diseases. Therefore, the authors are interested in whether gas plasma sterilization is useful for the inactivation of various “prions”, “prionoids”, or “prion-like aggregates” derived from humans and animals.

## Figures and Tables

**Table 2:** Survival of mice injected with prions treated using RENO-S130 (non-lumen and Eco mode) and untreated prions.

Treatment	*N*/*N*_0_ ^1^
Untreated	6/6
Non-lumen mode	1/6
Eco mode	1/6

^1^ *N*, number of dead animals; *N*_0_, number of inoculated animals. The inoculated mice were observed at 576 days post inoculation.

**Table 3:** Incubation times of mice injected with prions treated using RENO-S130 (non-lumen and Eco mode) and untreated prions.

Treatment	Incubation Times
Untreated	191.0 ± 9.0 days
Non-lumen mode	>576 days (401 days) ^1^
Eco mode	>576 days (181 days) ^1^

^1^ One of the mice injected with the non-lumen mode- and Eco mode-treated prions developed symptoms at the days post inoculation indicated in parentheses.

**Table 4:** Representative results of prion inactivation using gas plasma besides hydrogen peroxide gas plasma.

Prions	Instruments	Plasma Types (Total Process Time for the Treatment)	Sample Types	Main Results of Inactivation	Reference
Scrapie (Chandler)	Nitrogen gas plasma (BLP-TES)	Plasma generated by applying a short high-voltage pulse for a short time period using an IES circuit with an SI thyristor to nitrogen (30 min)	Prion-contaminated cover glass	Mean incubation time of animals prolonged to 251 days (plasma-treated) from 218 days (untreated)	[27]
Scrapie (RML5)	Air plasma (derived from HT2103 source)	Corona plasma (20 min)	Drops of prion-infected brain homogenate in well of microplate	Density of prion-staining in cell infectivity assay was reduced to less than 1/8 (1% prion-infected brain homogenate) and 1/30 (0.1% prion-infected brain homogenate) compared to untreated counterparts.	[28]
Scrapie (263 K)	Ar/O_2_ plasma	RF plasma (60 min)	Prion-contaminated steel wire	Survival rates of animals were 100% (mouse) after injection with Ar/O_2_ plasma-treated prions (steel wire). Survival of untreated control was 0%.	[29]
Scrapie (263K) and mouse-adapted BSE (6PB1)	Ar, Ar/O_2_, Ar/N_2_, Ar/N_2_/O_2_ -plasma (BIODECON ICP reactor)	Microwave-derived ICP (10 min)	Prion- contaminated steel wire or silk suture	Survival rates of animals were 50% (hamster) and 100% (mouse) after injection with Ar/O_2_ plasma-treated prions (steel wire). Survival of untreated control was 0%.	[30]

RML5: Rocky Mountain Laboratory scrapie strain 5; ICP: inductively coupled plasma; IES: induction energy storage; SI: static induction.

**Table 5:** Disease incubation time among mice injected with prion treated with nitrogen gas plasma instrument (BLP-TES) (30 min) and untreated prion (0 min).

Nitrogen Gas Plasma Treatment Time	Mean Incubation Time ± SEM ^1^
0 min	218.8 ± 3.2 days
30 min	251.3 ± 9.4 days ^2^

^1^ SEM, standard error of the mean. ^2^ *p* < 0.05. Reproduced from [27] and published under an open access Creative Commons CC BY 4.0 license.

## Data Availability

Not applicable.
